# Metabolism of alkenes and ketones by *Candida maltosa* and related yeasts

**DOI:** 10.1186/s13568-014-0075-2

**Published:** 2014-10-10

**Authors:** Andy Beier, Veronika Hahn, Uwe T Bornscheuer, Frieder Schauer

**Affiliations:** 1 Institute of Microbiology, Department of Applied Microbiology, Greifswald University, Friedrich-Ludwig-Jahn-Str. 15, Greifswald, 17487, Germany; 2 Institute of Biochemistry, Department of Biotechnology & Enzyme Catalysis, Greifswald University, Felix-Hausdorff-Str. 4, Greifswald, 17487, Germany

**Keywords:** Hydrocarbon, alkene, ketone, Candida, yeast, biotransformation

## Abstract

Knowledge is scarce about the degradation of ketones in yeasts. For bacteria a subterminal degradation of alkanes to ketones and their further metabolization has been described which always involved Baeyer-Villiger monooxygenases (BVMOs). In addition, the question has to be clarified whether alkenes are converted to ketones, in particular for the oil degrading yeast *Candida maltosa* little is known. In this study we show the degradation of the aliphatic ketone dodecane-2-one by *Candida maltosa* and the related yeasts *Candida tropicalis*, *Candida catenulata* and *Candida albicans* as well as *Trichosporon asahii* and *Yarrowia lipolytica*. One pathway is initiated by the formation of decyl acetate, resulting from a Baeyer-Villiger-oxidation of this ketone. Beyond this, an initial reduction to dodecane-2-ol by a keto reductase was clearly shown. In addition, two different ways to metabolize dodec-1-ene were proposed. One involved the formation of dodecane-2-one and the other one a conversion leading to carboxylic and dicarboxylic acids. Furthermore the induction of ketone degrading enzymes by dodecane-2-one and dodec-1-ene was shown. Interestingly, with dodecane no subterminal degradation products were detected and it did not induce any enzymes to convert dodecane-2-one.

## 1 Introduction

Aliphatic hydrocarbons (alkanes) are major components of natural gasoline and crude oil. However, alkanes can also be found in wax layers of plants and animals to reduce their loss of water by evaporation (Cheesbrough and Kolattukudy [1988]). These mostly very hydrophobic substances can be used as sole carbon and energy sources for microbial growth. For this, oxygen serves as electron acceptor under aerobic conditions, whereas under anaerobic conditions sulfate and nitrite have this function for some bacteria (Ji et al. [2013]). If oxygen is available, in most cases - especially in eukaryotic microorganisms - the degradation of alkanes is initiated by cytochrome P450 monooxygenases. The function of these enzymes is the oxidation of the *n*-alkanes to alcohols and the ω-hydroxylation of fatty acids (Scheller et al. [1998]). In course of the oxidation of aliphatic hydrocarbons by yeasts a mono- or diterminal degradation is possible (Blasig et al. [1988]; Käppeli [1986]; Mauersberger et al. [1996]; Schunck et al. [1987]). Within the monoterminal pathway the alkanes are converted by terminal hydroxylation to the corresponding primary alcohols, which are subsequently oxidized by alcohol dehydrogenases (or alcohol oxidases) and aldehyde dehydrogenases to fatty acids, which are further degraded via β-oxidation (Watkinson and Morgan [1991]). For diterminal degradation primary alcohols from the monoterminal pathway are hydroxylated at their ω-position resulting in the formation of diols. These are subsequently oxidized to ω-hydroxy fatty acids and then to dicarboxylic acids which are degraded by β-oxidation (Coon [2005]; Kester and Foster [1963]; Krauel et al. [1973]; Watkinson and Morgan [1991]). For some bacteria and filamentous fungi also a subterminal degradation is possible. Here, the initial hydroxylation happens intramolecularly and a secondary alcohol is formed (Forney and Markovetz [1970]). The secondary alcohol is oxidized to the corresponding ketone, subsequently converted to an ester by a Baeyer-Villiger monooxygenase (BVMO) which can be hydrolysed by an esterase to an alcohol and a fatty acid (Forney and Markovetz [1970]; Kirschner et al. [2007]; Kotani et al. [2007]; Lottmann et al. [1999]). After the primary oxidation of the alkanes in all cases efficient fatty acid oxidation systems as well as multiple anaplerotic metabolic sequences (e.g. acetyl-CoA metabolism, gluconeogenesis) are required (Schauer [2001]). In general it is claimed that a mono- and partly di- and subterminal degradation of hydrocarbons is performed by filamentous fungi (Rehm and Reiff [1981]). However, for yeast the subterminal degradation is unclear. Some yeasts possess excellent abilities to degrade *n*-alkanes and other aliphatic hydrocarbons. *Candida maltosa* is a yeast which is able to metabolize hydrocarbons fastly (Komagata et al. [1964]). Thus, its growth with *n*-hexadecane is faster compared to that with glucose. That is the reason why *C. maltosa* is sometimes called “super oil yeast” and cause of the assumption that its alkane metabolizing enzymes have a high catalytic activity. The transfer of *C. maltosa* from a medium with glucose to one with *n*-alkanes results in the induction of several catabolic enzymes and further proteins as well as changes in the endoplasmatic reticulum and amount of peroxisomes in which these enzymes and proteins are located. Furthermore the degradation of *n*-alkanes, primary alcohols, secondary alcohols and ketones can be increased in *C. maltosa* in varying degrees according to their respective chain length with the addition of biotin (Schauer [1988]). The availability of only short-chain hydrocarbons as carbon source results in a complete degradation to acetyl-CoA. This is amongst others used for the *de novo* fatty acid synthesis under requirement of biotin as cofactor for carboxylases (Tehlivets et al. [2007]). However in case that long-chain hydrocarbons are available as well, these can be converted to fatty acids directly and in conclusion no carboxylation and therefore no biotin is required (Schauer [1988]).

Unsaturated aliphatic hydrocarbons (alkenes) can be found in nature as gaseous and volatile compounds. Examples are the plant hormone ethene, isoprene from foliage and various monoterpenes as main components in plant oils (Abeles et al. [1992]; Primrose [1979]; Rasmussen [1970]). Alkenes have received some attention as oxidable substrates for microorganisms. Investigations about the metabolism of hexadec-1-ene in *Y. lipolytica* indicated the formation of the corresponding ω-unsaturated primary and secondary alcohols and the respective fatty acids by oxidation of the saturated end of this alkene. Moreover the oxidation of the double bond resulted in the conversion to 1,2-epoxyhexadecane, hexadecane-1-ol, hexadecane-2-ol, 2-hydroxyhexadecanoic acid and hexadecane-1,2-diol. The latter was converted to the 2-hydroxy acid and after that oxidatively decarboxylated to the one carbon atom shortened fatty acid (Bruyn [1954]; Klug [1969]; Klug and Markovetz [1967], [1968]; Stewart et al. [1960]).

Apart from the formation from alkanes and alkenes by microorganisms, ketones are occurring in plant oils, insects and mammals (Forney and Markovetz [1971]). Due to that ketones are common substances in the environment and therefore there are microorganisms that can convert those. Forney *et al.* showed the formation of undecane-1-ol and undecyl acetate from tridecane-2-one by *Pseudomonas multivorans* (Forney et al. [1967]). In the course of further degradation undecyl acetate was hydrolyzed to undecane-1-ol and acetate. Moreover, it was reported that the strain *Gordonia* spec. TY-5 can convert propane to propan-2-ol and further to acetone (Kotani et al. [2007]). This bacterium formed methyl acetate from the latter with a BVMO, which was subsequently hydrolyzed to methanol and acetate. This kind of ketone metabolism can not only be found in prokaryotes. One example from the fungus kingdom is the conversion of progesterone to testosterone acetate by *Cladosporium resinae* and the subsequent ester hydrolysis to the steroid alcohol and acetate (Fonken et al. [1960]; Rahim and Sih [1966]). Another one is the formation of ε-caprolactone from cyclohexanone by the cycloalkanone monooxygenase from *Cylindrocarpon radicicola* (Leipold et al. [2012]).

However, there are no studies about the metabolism of ketones in yeasts. One can only suppose that it might be the same as in other fungi and bacteria but experimental evidence is missing. To understand the metabolism of aliphatic ketones in yeasts *Candida maltosa*, *Candida albicans*, *Candida tropicalis*, *Candida catenulata*, *Yarrowia lipolytica* and *Trichosporon asahii* were cultivated in this work with dodecane-2-one as sole carbon and energy source. Furthermore it is unclear, whether ketones could be formed from alkanes or alkenes by the hydrocarbon oxidizing yeast *Candida maltosa*. To answer this question*, C. maltosa* was also cultivated with dodecane and dodec-1-ene to investigate their degradation pathways. In addition, resting cells of *C. maltosa* were incubated with dodecane-2-one, dodec-1-ene and dodecane after pre-cultivation with one of these substrates to show their conversion rates and the formation of products resulting from ketone degradation.

## 2 Materials and methods

### 2.1 Chemicals

All chemicals were purchased from Fluka (Buchs, Switzerland) or Sigma-Aldrich (Steinheim, Germany).

### 2.2 Yeast strains and culture conditions

Experiments were carried out using *Candida maltosa* SBUG 700*, Candida tropicalis* SBUG 1019*, Candida catenulata* SBUG 512*, Yarrowia lipolytica* SBUG 1888*, Trichosporon asahii* SBUG 833 *and Candida albicans* SBUG 5121 which are deposited at the strain collection of the Department of Biology of the University of Greifswald (SBUG) from where they can be obtained upon request. Cultures were inoculated from an overnight malt agar culture.

Yeast cells were cultivated in a mineral salt medium (5 g NH_4_H_2_PO_4_, 2.5 g KH_2_PO_4_, 1 g MgSO_4_ × 7 H_2_O, 0.02 g Ca(NO_3_)_2_ × 7 H_2_O, 2.0 mg FeCl_3_ × 6 H_2_O, 0.5 mg H_3_BO_3_, 0.4 mg MnSO_4_ × 5 H_2_O, 0.4 mg ZnSO_4_ × 7 H_2_O, 0.2 mg Na_2_MoO_4_, 0.1 mg CuSO_4_ × 5 H_2_O, 0.1 mg CoCl_2_, 0.1 mg KI in 1 L dest. H_2_O, pH 5.4 (Hornei et al. [1972])) supplemented with 1% (v/v) vitamine solution (Van der Walt and van Kerken [1961]) and 1% (v/v) of the respective substrate (dodecane-2-one, dodec-1-ene, dodecane). Cultures grew until an OD_600nm_ of 3 at 30°C and 250 rpm for 31.5 h (dodecane), 32 h (dodec-1-ene), 40 h (dodecane-2-one, *C. maltosa*), 290 h (*C. albicans*), 72 h (*C. catenulata*) 47 h (*C. tropicalis*), 189 h (*Y. lipolytica*) and 381 h (*T. asahii*). Cells were centrifuged at 4°C and 10000 × g for 15 min (Sorvall RC-5B Plus Refrigerated Superspeed Centrifuge). The supernatant was analyzed for secreted products (see section Liquid-liquid extraction). Controls contained either 1% (w/v) glucose, no substrate or no cells.

### 2.3 Preparation of resting *Candida maltosa* cells

Cell pellets from cultures of *Candida maltosa* grown with dodecane, dodec-1-ene or dodecane-2-one were washed three times with 100 mL sodium phosphate buffer (SPB, 67 mM, pH 5.4). Subsequently cell suspensions with an OD_600nm_ of 5 (approx. 5.8*10^7^ cfu/ml, total cell count of 2.4*10^9^ cells/ml, cell dry mass of 3.49 mg/ml) were prepared in SPB.

### 2.4 Biotransformations

For biotransformation experiments, resting cell preparations of *Candida maltosa* were used. Reactions were carried out in SPB at 0.25% (v/v) substrate concentration. Cells were centrifuged and suspended in SPB. The OD_600nm_ was adjusted to 5. No vitamines/cofactors were added. Cell suspensions were used in amounts of 2 mL. Controls contained no cells or no substrate. The total volume in every case was 10 mL. Incubation was performed in 500 mL flasks at 30°C and 250 rpm for 0, 1 and 4 hours. After these times the whole biotransformation approach of 10 mL was centrifuged for 5 min at 3857 g.

### 2.5 Liquid-liquid extraction

Before extraction the pH-value of the supernatants was first adjusted to pH 9 by addition of 25% NaOH (v/v). Extraction of samples was achieved by vortexing three times with 20 mL of diethyl ether (basic extracts). Next, the pH-value was set to pH 2 by addition of 32% HCl (v/v). Then, samples were extracted again as mentioned above (acidic extracts). Samples were dried over anhydrous sodium sulphate, concentrated in a rotary evaporator and desiccated with nitrogen. For GC/MS analysis the samples were dissolved in 500 μl hexane. The acidic extracts were derivatized by methylation using diazomethane (De Boer and Backer [1956]). For investigation of metabolites formed during cultivation the respective supernatants were extracted three times with 50 mL diethyl ether first at pH 9 and then at pH 2. Further workflow was the same as mentioned above.

### 2.6 Structure elucidation of products by GC/MS

GC/MS analyses were performed on an Agilent gas chromatograph 7890A GC system (Waldbronn, Germany) equipped with a 30 m HP-5 ms column (0.25 mm by 0.25 μm film) and linked to a mass selective detector 5975C inert XL EI/CI MSD with a quadrupole mass spectrometer.

For separation of products a temperature program was used, starting with 5 min at 60°C followed by a ramp from 60–120°C at 20°C/min. The 120°C were maintained for 5 min and then followed by heating the column to 200°C at 3°C/min, to 280°C at 20°C/min and lastly 5 min at 280°C. For quantification of products formed commercially available standard substances were measured with concentrations from 0.5-10 mM for the creation of calibration curves.

Analytical data of the products can be found in the Additional file 1: Table S1.

## 3 Results

### 3.1 Biotransformation of dodecane-2-one, dodec-1-ene and dodecane by growing yeast cells

**Figure 1:**
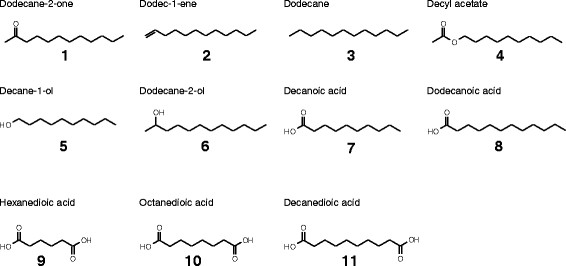
**Substrates (1, 2, 3) and extracellular metabolites (1, 4–11) detected in cultures containing either dodecane-2-one, dodec-1-ene or dodecane.** Carboxylic acids were detected as methyl esters.

All the investigated yeasts *C. maltosa*, *C. albicans*, *C. catenulata*, *C. tropicalis*, *Y. lipolytica* and *T. asahii* and were able to grow with dodecane-2-one (**1**) as a sole source of carbon and energy. In the supernatants of the culture media a variety of metabolites was formed (Figure 1, see Additional file 1: Figure S1 for an exemplary GC chromatogram). The spectrum of products formed from **1** was very similar among the test organisms whereas the amount of formed products differed (Table 1). However, in every case dodecane-2-ol (**6**) and also (with the exception of *C. albicans*) decyl acetate (**4**) was detected.

**Table 1:** **Metabolites identified in the yeast culture supernatants after growth until OD**_**600nm**_**of 3 with 1% dodecane-2-one (1), dodec-1-ene (2) or dodecane (3) as sole carbon and energy source at 30°C and 250 rpm**

**Strain**	**Substrate**	**Detected metabolites**
*C. maltosa*	**1**	**4, 5, 6, 9, 10, 11**
	**2**	**1, 6, 7, 8, 9**
	**3**	**8, 9**
*C. albicans*	**1**	**6**
*C. tropicalis*	**1**	**4, 5, 6, 9, 10, 11**
*C. catenulata*	**1**	**4, 5, 6, 9, 10, 11**
Y. lipolytica	**1**	**4, 5, 6, 9, 10**
*T. asahii*	**1**	**4, 5, 6, 9, 11**

In contrast to the incubation of *C. maltosa* with **1** in experiments with dodec-1-ene (**2**), only five were detected (Table 1). In cultures with dodecane (**3**) only two metabolites were formed, whereas it has to be highlighted that here in contrast to **2** no formation of ketones, esters and secondary alcohols could be observed. In control appoaches with glucose, no substrate or no cells none of these substances could be detected.

### 3.2 Biotransformation of dodecane, dodec-1-ene and dodecane-2-one by resting *C. maltosa* cells

Differences in the induction of enzymes involved in ketone metabolism, including BVMOs, in *C. maltosa* after cultivation with **1**, **2** and **3** as sole carbon and energy source were examined. Therefore the conversion of the substrates **1**, **2** and **3** (see section Conversion rate of **1**–**3** in dependence of the pre-culture substrate) and the formation of compounds (see section Product formation in course of biotransformation) were analysed by GC/MS.

### 3.3 Conversion rate of 1–3 in dependence of the pre-culture substrate

**Figure 2:**
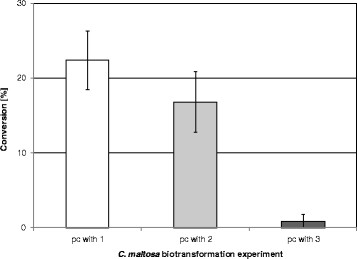
**Conversion of dodecane-2-one (1) after 0.5 h by resting cells of** ***C. maltosa*** **either pre-cultured (pc) with 1, dodec-1-ene (2) or dodecane (3) for induction followed by biotransformation (bt).** (For determination of conversion GC/MS was used).

On the basis of the determined data the conversion of the substrates was calculated (Figure 2). The conversion started immediately with a high rate. Thus, approximately 20% were transformed in almost every sample within 30 min. Cells pre-cultured with **1** transformed 25.18 μmol (22.4%) of **1**. The conversion with cells cultivated with **2** was comparable to that. In this case 18.92 μmol (16.8%) of **1** were transformed. Cells cultured with **3** showed negligible conversion of **1** (1.03 μmol, 0.9%). Moreover, the conversion of **2** was 24.08 μmol (21.4%) and 24.58 μmol (21.8%) with cells grown on **2** and **3**, respectively (data not shown). Apart from that the conversion of **3** with cells pre-cultured with **3** was 18.07 μmol (16.4%) (data not shown).

### 3.4 Product formation in course of biotransformation

**Figure 3:**
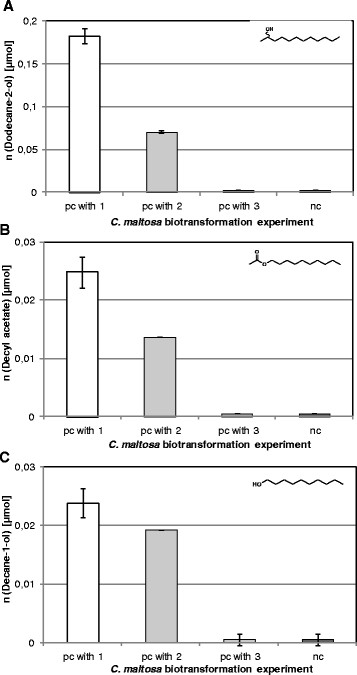
**Formation of dodecane-2-ol (A), decyl acetate (B) and decane-1-ol (C) from dodecane-2-one (1) after 0.5 h by resting cells of** ***C. maltosa*, pre-cultured (pc) with 1, 2 or 3 as well as by resting cells of the hydrocarbon non-utilising yeast** ***Candida utilis*, pc with glucose (=negative control, nc).**

In case of *C. maltosa* cells incubated with 112.3 μmol **1**, which were pre-cultured with **1** or **2**, the metabolites **4**, **5** and **6** were detected (Figure 3). Cells grown on **3** did not secret these three compounds. Differences in product concentration were observed for **6** (Figure 3A). Thus, cells grown on **1** formed 0.18 μmol **6** while pre-cultured with **2** only 0.07 μmol were formed. The formation of **4** from **1** also differed in dependence of the substrate used for pre-cultivation (Figure 3B). Only cells grown on **1** were able to secrete an amount of 0.03 μmol **4**. Biotransformations with cells cultivated on **2** resulted in the formation of approx. 0.02 μmol **4**. The primary alcohol **5**, the decomposition product of **4**, was formed in similar rates as **4** (Figure 3C). Thus, amounts of **5** of approx. 0.02 μmol were detected with cells pre-cultured on **1** and **2**. In control experiments with *C. utilis*, a yeast which is not able to metabolize aliphatic hydrocarbons, and **1** as substrate no product formation was observed. In addition, after incubation of *C. maltosa* cells pre-cultured with **2** or **3** and the biotransformation substrates **2** and **3** neither **4**, **5** nor **6** were detected (data not shown). In approaches without cells or substrate theses metabolites could not be detected.

## 4 Discussion

In this study we investigated the metabolism of alkenes and ketones in yeasts. We were able to detect the degradation products **1**, **6**, **7**, **8** and **9** from dodec-1-ene and **4**, **5**, **6**, **9**, **10** and **11** from dodecane-2-one in cultures of growing yeast cells and partially as well in biotransformation approaches with resting yeast cells. The production of some of these metabolites provided evidence of the induction of ketone degrading enzymes after growth on **1** or **2**, but not on **3**, in the investigated hydrocarbon oxidizing yeasts.

### 4.1 Biotransformation of dodecane-2-one by growing yeast cells

After cultivation of *C. maltosa*, *C. tropicalis*, *C. catenulata*, *C. albicans*, *Y. lipolytica* und *T. asahii* with **1** or **2** as sole carbon and energy source the culture media were used for the determination of formed metabolites to study the metabolism of aliphatic ketones and alkenes.

**Figure 4:**
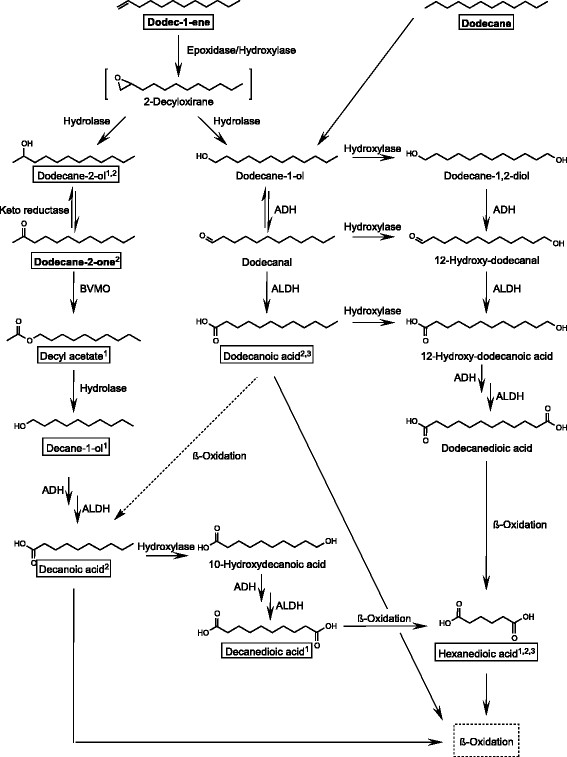
**Suggested metabolism of dodecane-2-one, dodec-1-ene and dodecane in** ***Candida maltosa*** **SBUG 700.** A black frame indicates intermediates detected from cultures of *C. maltosa* with **1**, **2** or **3** as sole carbon and energy source. Metabolites found in cultures 1: with **1**, 2: with **2**, 3: with **3**, ADH: Alcohol dehydrogenase, ALDH: Aldehyde dehydrogenase

In culture media from *C. maltosa* with **1** as substrate in total six metabolites were detected. Although in quantity less compounds were secreted by some other yeasts, the products were the same. The conversion of **1** was either initiated by a keto reductase or a BVMO (Figure 4).

The first degradation pathway was identified for all investigated yeast strains with exception of *C. albicans* which is initiated by a BVMO with the formation of **4**. The enzymatic Baeyer-Villiger oxidation can lead to both regioisomeric products of the corresponding ketone (Alphand et al. [1989]; Balke et al. [2012]; Kelly et al. [1995]; Leisch et al. [2011]; Rehdorf et al. [2010]; Song et al. [2013]). In this case the “normal” product is **4** and undecanoic acid methyl ester would be the “abnormal” one. It seems that *C. maltosa* as a whole cell catalyst rather formed the “normal” product **4** than the “abnormal” undecanoic acid methyl ester as nothing of the latter could be detected. The prefered formation of the “normal” product from a ketone by a BVMO has been described already (Mihovilovic and Kapitán [2004]). In addition, **5** was detected which was probably formed by conversion of **4** via a hydrolase. A similar reaction was reported by Forney *et al.* in which *Pseudomonas multivorans* converted tridecane-2-one to undecyl acetate and subsequently hydrolysed it to undecane-1-ol and acetate (Forney and Markovetz [1968]; Forney et al. [1967]). Due to the slow growth of *C. albicans* with **1** the accumulation of products may not have been sufficient to detect them in the cultures of this yeast. That could be the reason why just **6** was found as a metabolite.

In addition, the formed decanoic acid was probably hydroxylated at its ω-end resulting in the formation of 10-hydroxydecanoic acid which was further oxidized to the respective dicarboxylic acid decanedioic acid (**11**). Decandioic acid was degraded by β-oxidation which was confirmed by (De Boer and Backer [1956]) detection of its C_2_ shortened intermediates until hexandioic acid. Therefore a diterminal degradation took place what has been previously reported for the filamentious fungus *Mortierella isabellina*, the bacteria *Rhodococcus rhodochrous* and *Corynebacterium* spp. as well as for the yeasts *Candida guilliermondii* and *Candida* spp. (Coon [2005]; Kester and Foster [1963]; Krauel et al. [1973]; Watkinson and Morgan [1991]). At least some of the intermediates of the β-oxidation of this dicarboxylic acid were detected in the cultures of all investigated yeasts. The formation of dicarboxylic acids ranging from six to 12 carbons from dodecane for *C. tropicalis* and from 12 to 16 carbons from the respective alkanes for *Y. lipolytica* has already been reported (Picataggio et al. [1992]; Smit et al. [2005]).

Another degradation pathway is proposed by the detection of **6**. Therefore the yeasts have to contain at least one keto reductase that can reduce **1** to this secondary alcohol. The reduction of ketones like hexane-2-one, methyl acetoacetate and α-tetralone to the respective alcohols was described for other yeasts such as *Saccharomyces cerevisiae* and *Candida viswanathii* (Patil et al. [2013]; Wolfson et al. [2013]). It was assumed that this keto reductase has a relatively high activity due to the 7.4-fold and 7.7-fold higher amount of detected **6** in comparison to **4** and **5** respectively in approaches of **1** pre-cultured resting cells incubated with **1**.

### 4.2 Biotransformation of dodecane-1-ene and dodecane by growing yeast cells

In *C. maltosa* cultures with **2** as sole source of carbon and energy five compounds were detected. One of the products was **1**. For the formation of this compound **2** reacted possibly via 1,2-epoxidodecane to **6** and subsequently to **1** (Figure 4).

In addition, dodecane-1-ol possibly was formed also via 1,2-epoxidodecane from **2** and oxidized to dodecanal and subsequently to **8**. This acid was further metabolized via β-oxidation. Some of these reactions were already described for *Y. lipolytica*, *C. tropicalis* and *Candida* spec. (Bruyn [1954]; Klug [1969]; Klug and Markovetz [1967], [1968]; Terasawa and Takahashi [1981]). By ω-oxidation of **8** the dioic acid was formed and transformed by β-oxidation to **9**. The reason why there was no formation of dodecane-1-ol from **2** detectable could be that this primary alcohol was metabolized too fast to **7** and the subsequent products. Also, *C. tropicalis* and *Y. lipolytica* were able to form tetradecane-2-ol and tetradecane-2-one from tetradec-1-ene (Terasawa and Takahashi [1981]).

In contrast, in *C. maltosa* culture media with **3** neither **4**, **5** nor **6** was formed. Instead, the degradation was accomplished by a monoterminal oxidation. Thus, dodecanoic acid was detected. In addition, a diterminal oxidation was observed supported by the detection of hexandioic acid. A similar study with *Candida rugosa* and decane indicated a mono- and diterminal oxidation as well (Mizuno et al. [1966]).

### 4.3 Dependence of the enzyme induction on the substrate used for pre-cultivation

In order to reproduce the differences in product formation after cultivation of alkanes and alkenes the induction of ketone converting enzymes was studied and biotransformations with resting cells of *C. maltosa* SBUG 700 were performed. Cells grown on either **1**, **2** or **3** were examined regarding substrate conversion and product formation. Our results are matching the principle of the simultaneous adaptation by Stanier ([1947]). According to that the catabolism of all occurring intermediates is induced by incubation with the initial substrate if the participating enzymes of that pathway are inducible. In agreement with this principle **1** was immediately converted after pre-cultivation with **2** although the conversion rate was slightly reduced compared with cells grown with **1** (Figure 2). In contrast, cells pre-cultured with **3** transformed **1** just about 0.91% probably due to the lack of conversion of **3** to **1** during pre-cultivation. In addition, an enzyme with BVMO activity and a keto reductase were induced in cells pre-cultured with **1** or **2** as those formed **4** and its fission product **5** as well as **6** after incubation with **1** (Figure 3). The fact that with **1** pre-cultured cells formed higher amounts of all these 3 products than with **2** pre-cultured cells (e.g. 0.18 vs. 0.07 μmol dodecane-2-ol) indicates that the presence of the ketone leads to a stronger induction. Thus, the conversion of **2** to **1** during cultivation was probably not sufficient enough to form an adequate amount of **1** to induce the expression of ketone degrading enzymes as strong as with pure **1** alone. On **3** pre-cultured cells incubated with **1** formed none of the compounds **4**, **5** and **6**. Thus, the above mentioned lack of conversion of **3** to **1** during pre-cultivation was confirmed. This is leading to the conclusion that a subterminal oxidation of alkanes, in contrast to alkenes, does not take place in *C. maltosa*. Impurities of alkenes in alkane preparations can therefore lead to incorrect interpretations of a participation of subterminal reactions.

It was shown that *C. maltosa* can convert dodec-1-ene to dodecane-2-one and this aliphatic ketone to decyl acetate resulting from a BVMO activity which can also be found in all of the here investigated hydrocarbon oxidizing yeasts. The reduction of **1** to its corresponding secondary alcohol **6** was additionally shown.

In conclusion, the ketone metabolism in yeasts is comparable to that of bacteria. Ketones can either be oxygenated to esters or be reduced to the corresponding alcohol. These pathways seem to be quite ubiquitary among several hydrocarbon oxidizing yeasts. *C. albicans* was the only yeast not being able to form an ester out of the ketone in a detectable amount. Beyond this, alkenes can be converted to ketones and thus induce the production of ketone-degrading enzymes whereas this way seems to be invalid for alkanes.

## Competing interests

The authors declare that they have no competing interests

## Authors’ contributions

FS and UB initiated the project. AB performed the experiments with support by VH. All authors analyzed the data, wrote and approved the final manuscript.

## Additional file

## Supplementary Material

Additional file 1: Table S1. Overview of extracellular compounds detected in culture media with dodecane-2-one or dodec-1-ene as sole source of carbon and energy. Click here for file
