# Supplementary material for: Metabolism of alkenes and ketones by Candida maltosa and related yeasts
Source: AMB Express. 2014 Oct 10;4:75. doi: 10.1186/s13568-014-0075-2 (PMC4192553; doi:10.1186/s13568-014-0075-2)
Supplement: Additional file 1: Table S1. — Overview of extracellular compounds detected in culture media with dodecane-2-one or dodec-1-ene as sole source of carbon and energy. [file s13568-014-0075-2-S1.docx]

Supplementary Material

Metabolism of alkenes and ketones by *Candida maltosa* and related yeasts

Andy Beier^1,2^, Veronika Hahn^1^, Uwe T. Bornscheuer^2*^, Frieder Schauer^1^

^1^Institute of Microbiology, Dept. of Applied Microbiology, Greifswald University, Friedrich-Ludwig-Jahn-Str. 15, 17487 Greifswald, Germany

^2^Institute of Biochemistry, Dept. of Biotechnology & Enzyme Catalysis, Greifswald University, Felix-Hausdorff-Str. 4, 17487 Greifswald, Germany

^*^Correspondence: U. T. Bornscheuer, Institute of Biochemistry, Dept. of Biotechnology & Enzyme Catalysis, Greifswald University, Felix-Hausdorff-Str. 4, 17487 Greifswald, Germany. Tel: +49-3834-864367 Fax: +49-3834-86-794367

E-mail: [uwe.bornscheuer@uni-greifswald.de](mailto:uwe.bornscheuer@uni-greifswald.de)

Table S1. Overview of extracellular compounds detected in culture media with dodecan-2-one or dodec-1-ene as sole source of carbon and energy.

| **No.** | **Compound**  **(MW [g/mol])** | **R_t_ [min]** | **Fragmentation m/z,**  **relative intensity [%]** |
| --- | --- | --- | --- |
| 1 | Dodecan-2-one^1^  (184) | 15,836 | 39 [5], 41,05 [18], 41,95 [5], 43 [65], 55 [14], 56,05 [5], 57,05 [13], 57,95 [100], 59 [35], 67 [4], 69 [6], 70,05 [4], 70,95 [44], 72 [3], 81,05 [4], 82,1 [7], 83,05 [5], 84,05 [4], 85,05 [15], 95,05 [6], 96,05 [7], 97,05 [6], 98,05 [3], 113,05 [3], 124,05 [7], 126,1 [9], 127,1 [4], 169,1 [4], 184,2 [9] |
| 2 | Dodec-1-ene^2^  (168) | 10,105 | 39,1 [26], 41,05 [86], 42,05 [22], 43,05 [71], 53,1 [11], 54,05 [16], 55,1 [100], 56,05 [77], 57,05 [55], 67,1 [18], 68,1 [15], 69,05 [82], 70,1 [73], 71,1 [23], 81,1 [6], 82,1 [17], 83,1 [65], 84,1 [41], 85,1 [12], 96,1 [7], 97,1 [49], 98,1 [20], 110,1 [3], 111,1 [16], 112,1 [7], 125,1 [6], 126,1 [3], 140,05 [4], 168,1 [9], |
| 3 | Dodecane  (170) | 10,264 | 39,1 [7], 41,1 [34], 42,1 [9], 43,1 [62], 55,1 [17], 56,1 [17], 57,1 [100], 58,1 [4], 69,1 [8], 70,1 [15], 71,1 [66], 72,1 [4], 83,1 [4], 84,1 [9], 85,1 [41], 86,1 [3], 98,1 [8], 99,1 [8], 112,1 [5], 113,1 [5], 127,1 [4], 170,2 [6] |
| 4 | Decyl acetate^1^  (200) | 16,379 | 39 [6], 41 [31], 42 [14], 43 [100], 44 [4], 53 [3], 54 [5], 55 [43], 56 [38], 57 [22], 58 [4], 61 [32], 67 [7], 68 [12], 69 [35], 70 [43], 71 [10], 73 [6], 81,1 [3], 82 [13], 83 [33], 84 [21], 85,1 [6], 96 [4], 97,1 [22], 98 [12], 111 [12], 112,1 [13], 116 [4], 140,1 [4] |
| 5 | Decan-1-ol^1^  (158) | 11,860 | 39 [18], 40 [6], 41 [76], 42,1 [30], 43 [75], 44 [13], 45 [3], 53 [7], 54 [10], 55 [100], 56 [80], 57 [44], 67 [13], 68 [28], 69,1 [72], 70 [86], 71 [15], 73 [3], 81 [4], 82 [23], 83 [63], 84 [40], 85 [7], 96 [6], 97 [30], 98 [11], 111,1 [13], 112 [20], 125 [6] |
| 6 | Dodecan-2-ol^1^  (186) | 16,176 | 39 [4], 41 [19], 42 [6], 43 [23], 43,95 [6], 45 [100], 55 [23], 56 [12], 57 [21], 58 [3], 67 [3], 69 [19], 70 [12], 71 [11], 82 [4], 83 [17], 84,05 [9], 85,10 [6], 97,05 [19], 98,1 [6], 111,05 [8], 112,05 [3], 125,1 [3], 140 [4] |
| 7 | Decanoic acid^1^  (186) | 13,381 | 39 [4], 41 [14], 42 [4], 43 [17], 55 [17], 56 [3], 57 [6], 59 [10], 69 [8], 71 [3], 74 [100], 75 [10], 83 [4], 84 [3], 87 [54], 88 [5], 97 [3], 100,95 [7], 128,95 [4], 143,05 [15], 155,05 [8], 157,05 [3] |
| 8 | Hexanedioic acid^1^  (174) | 11,064 | 39 [12], 41 [24], 42 [17], 43 [31], 44 [3], 44,95 [7], 52,95 [5], 53,95 [8], 55 [80], 55,95 [10], 57 [4], 58 [7], 59 [100], 59,95 [4], 67,95 [4], 69 [8], 71 [5], 71,95 [8], 72,95 [30], 73,95 [43], 82 [12], 83 [24], 84 [4], 85 [5], 86,95 [13], 88,05 [3], 97 [4], 99 [3], 100,95 [65], 103 [3], 110 [3], 111 [60], 112 [5], 114 [82], 114,95 [16], 141,95 [11], 142,95 [46], 144,05 [4] |
| 9 | Octanedioic acid^1^  (202) | 17,628 | 39 [20], 39,95 [3], 41 [56], 42 [16], 43 [51], 44 [3], 45 [10], 53 [8], 54 [6], 55 [100], 55,95 [23], 57 [12], 57,9 [4], 58,95 [79], 67 [14], 67,95 [23], 69 [98], 69,95 [11], 71 [6], 71,95 [9], 73 [9], 73,95 [96], 74,95 [6], 81 [11], 82 [17], 83 [51], 83,95 [12], 85 [7], 86,95 [46], 87,95 [4], 93 [4], 96 [5], 96,95 [87], 97,95 [6], 98,9 [3], 100,95 [10], 110 [22], 111 [27], 112 [6], 112,95 [18], 114 [12], 116 [3], 126,9 [4], 127,95 [4], 129 [91], 129,95 [8], 136,9 [3], 138 [83], 139 [18], 140 [6], 140,95 [17], 142 [11], 170 [3], 171 [54], 172 [5] |
| 10 | Decanedioic acid^1^  (230) | 25,210 | 39 [12], 40 [3], 41 [44], 41,95 [15], 43 [37], 44 [3], 45 [7], 52,95 [6], 54 [5], 55 [100], 56 [12], 57 [8], 58,95 [55], 67 [13], 68 [6], 69 [38], 69,95 [6], 71 [4], 73,05 [10], 74 [93], 75 [6], 79 [7], 79,95 [5], 81 [14], 81,95 [6], 83 [39], 84 [46], 85 [6], 86,95 [34], 87,95 [4], 93 [4], 94 [4], 95 [9], 96 [12], 97 [48], 98 [65], 99 [5], 100,95 [9], 107 [5], 109 [3], 109,95 [8], 111 [10], 112 [6], 114,95 [8], 119,95 [3], 121 [10], 122,95 [3], 123,95 [9], 125 [60], 126 [5], 137 [3], 138 [35], 138,95 [13], 143,95 [4], 148 [5], 157,05 [29], 157,95 [3], 166 [25], 167 [3], 170 [4], 199,1 [36], 200,1 [4] |

MS-data of *C. maltosa* cultures 1: with dodecan-2-one (1), 2: with dodec-1-ene (2)

carboxylic acids were detected as methyl esters due to a previous derivatisation

Fig. S1. GC-chromatogram of an alkaline extract of a culture with *C. maltosa* and 1.
